# Perceptual Contaminants to Assessments of Visuospatial Working Memory: The Influences of Path Clutterness and Endpoint Crowding

**DOI:** 10.5334/joc.517

**Published:** 2026-08-25

**Authors:** Zechun Zhao, Freya Joessel, C. Shawn Green, Susanne M. Jaeggi, Aaron R. Seitz

**Affiliations:** 1 Department of Psychology, Northeastern University, Boston, MA, USA; 2 Department of Psychology, University of Wisconsin-Madison, Madison, WI, USA

**Keywords:** Visuospatial working memory, Perception, Corsi block tapping task

## Abstract

Identifying individual differences in various cognitive constructs has important implications for settings such as clinical and educational assessments. Yet, cognitive tasks themselves often do not measure a single construct. As such, there is value in understanding the mixture of constructs involved in cognitive task performance, as this would allow target constructs to be better isolated. For example, tasks assessing visuospatial working memory (VSWM), in addition to measuring the “of interest” processes of encoding and maintenance, also involve lower-level perceptual processes. Here, we examined perceptual contaminant effects on VSWM using a modified Corsi Block Tapping Task, in which participants observed and reproduced sequences of spatial locations. Two forms of perceptual interference were manipulated: path clutterness, in which distractors appeared near the virtual path connecting successive targets, and endpoint crowding, in which distractors appeared near the target locations themselves. Endpoint crowding produced clear decrements in task performance, whereas path clutterness produced no detectable changes in performance. These findings indicate that perceptual processes, specifically visual crowding, influence VSWM assessment. This underscores the importance of accounting for perceptual processes in the design and interpretation of working memory measures.

## 1. Introduction

Visuospatial Working Memory (VSWM) is commonly understood as the ability to temporarily store and manipulate visual and spatial information (Baddeley & Hitch, 1974). As a cognitive function, VSWM facilitates many daily activities, from driving cars, which involves paying attention to, encoding, retrieving, and predicting the locations of moving traffic (Zhang et al., 2024), to solving math problems by forming and manipulating visual representations of geometry (Bizzaro et al., 2018). Given its importance, numerous tasks have been developed to accurately assess VSWM. Here, we examine the challenge of measurement impurity in VSWM tasks, specifically in cases where the visual properties of stimulus displays may significantly contaminate estimates of VSWM capacity if those properties are not carefully controlled.

Specifically, we examine this in the context of the Corsi Block Tapping Task (CBTT), which is a widely used measure of VSWM in both experimental and clinical neuropsychology settings (Corsi, 1972; Milner, 1971). The original version of this task was administered via a 23 × 28 cm (9 × 11 inch) physical wooden board with 9 identical blocks (3 × 3 cm/~1.18 × 1.18 inch). An experimenter pointed to blocks in a sequence one by one, and when done with the full sequence, the participant was required to replicate the sequence. Sequence length increased progressively until the participant was no longer able to replicate the sequence accurately, with VSWM capacity being estimated as the length of the longest sequence the participant could correctly replicate (Berch, Krikorian & Huha, 1998).

Recent modified versions of the CBTT have introduced both structural (stimulus design) and procedural (test administration) variations to the original design, including changes in the total number of blocks (Wechsler, 1997), modality (Röser, Hardiess & Mallot, 2016; Ruggiero & Ichini, 2010; Piccardi et al., 2008), presentation of sequences (Siddi et al., 2020; Berch, Krikorian & Huha, 1998), and a transition from physical boards to digital devices (Brunetti, Del Gatto & Delogu, 2014). Despite these alterations from the original task, most variants of the CBTT share the underlying assumption that, within the subset of sequences presented to the participant, sequences with the same length have the same difficulty and therefore put a similar load on VSWM. However, memory span estimates from the CBTT are often not equivalent when the layouts of visuospatial items between assessments are distinct. For example, Ginsberg and colleagues (2017) noted that performance can vary widely at a fixed sequence length by showing that one can design sequences with a span of eight where performance will be almost 100% or as low as 6%. This inevitably leads to the threat that “various instantiations” of the same WM span measure may lead to different estimates of VSWM (Conway et al., 2005). A key issue as new implementations of CBTT are developed (Zsebi et al., 2023; Cochrane & Green, 2021; Nori et al., 2015), or more broadly as new VSWM tasks are designed, is that it is necessary to better understand the design elements that may differentially influence underlying cognitive processes and influence estimates of VSWM.

Importantly, VSWM is a multi-dimensional cognitive construct. The distributed WM network suggests that different brain regions contribute to the VSWM process with the general nature of their representations (Christophel et al., 2017). For example, under the sensory recruitment model of visual working memory, the early visual cortex (V1), which is assumed to be specialized in perception, is also activated during visual information maintenance in addition to the frontal and parietal areas (Adam, Rademaker & Serences, 2022; Serences, 2016). Perception acts as a filter in the feedforward encoding and determines the quality of information that memory stores and operates upon (van Lamsweerde & Johnson, 2017; van de Ven, Jacobs & Sack, 2012). As feedback, working memory also biases attention selection to the sensory information that matches the stored items (Gayet et al., 2017). Further, resource models of working memory suggest that selective attention can be unevenly and dynamically allocated, leading to some visual items being represented in WM more noisily and others more precisely (Oberauer, 2019; Souza et al., 2014; Bays & Husain, 2008). Critically, these models describe a complex interplay between working memory, visual attention and perceptual processes and suggest that low-level features are likely to influence VSWM performance.

A key example is that sensory load modulates span size (Ginsberg, Rinehart & Fielding, 2017; Parmentier, Elford & Maybery, 2005; Alvarez & Cavanagh, 2004; Orsini, Simonetta & Marmorato, 2004; Orsini et al., 2001), and this interference has been shown to involve early visual cortex (V1) (Yörük & Tamber-Rosenau, 2022). For example, in the CBTT, path crossings (defined as when a sequence path intersects itself), angularity (defined as the angle formed by consecutive paths connecting target blocks), and the physical length of the spatial path in sequences contribute significantly to the task difficulty (Orsini, Simonetta & Marmorato, 2004; Parmentier, Elford & Maybery, 2005). Similarly, Ginsberg and colleagues (2017) defined clutterness between two consecutive blocks in a spatial sequence as cases where distractor blocks overlap with the area formed by connecting the diagnostic vertex of the two target blocks. When more distractor blocks are in the clutter zone, more effort is required to discriminate them from the target blocks, and the precision of sequence replication is reduced.

### 1.1. The Present Study

**Figure 1:**
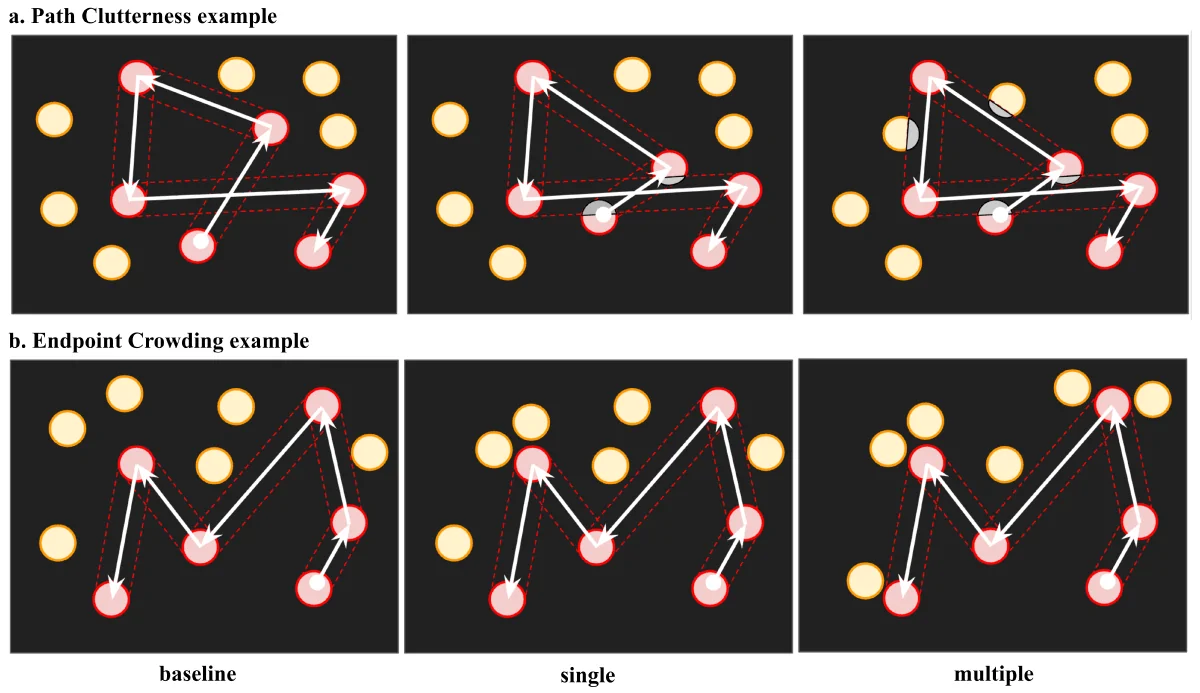
Example of **(a)** Path Clutterness and **(b)** Endpoint Crowding each with three levels (baseline, single, multiple). ***Note***. Sets of sequence examples used in the experiment. Pink circles are targets (can also be used to form clutterness/crowding) in the sequence; Yellow circles are distractors only (not part of the sequence). Solid white lines with an arrow show the direction of the sequence. Red dashed lines show the clutter zone of each sequence path. The grayed area in each circle shows overlap with the clutter zone. Note that all lines are invisible, and there is no color differentiation between circles during the actual experiment. **(a). Path Clutterness:** Only the 4th path is cluttered in the single level; Multiple paths are cluttered in the multiple level. **(b). Endpoint Crowding:** Only the 5th circle’s location is crowded in the single level; Multiple circles’ locations are crowded in the multiple level.

One challenge shown in the previous work on this topic is that the perceptual spatial features that can impact performance in a sequence of the CBTT are often confounded with each other. As such, separating their independent respective effects requires experimental care. In the present study, we clarify the influence of low-level visual features on the CBTT by examining the unique contributions of both path clutterness (Figure 1a) and endpoint crowding (Figure 1b) while controlling the variation in other features (e.g., number of path crossings, path length, etc). We operationalize clutterness as the proximity of blocks to paths connecting two points in the CBTT (Ginsberg, Rinehart & Fielding, 2017), while crowding is operationalized by the proximity of blocks to the endpoint target blocks of that path (Whitney & Levi, 2011; Levi, 2008; Polat et al., 2004). Based on the sensory recruitment and resource models of VSWM, we hypothesize that both clutterness and crowding will impair performance by altering visual input during encoding and increasing competition for limited working memory resources. Although crowding has been examined within the sensory recruitment framework using array-identification tasks (Yörük & Tamber-Rosenau, 2022), it has not been investigated in a VSWM task such as the CBTT. Furthermore, neither crowding nor clutterness has been systematically examined while controlling for other task-demand features such as path crossings and set size. To address these issues, the current study sought to directly quantify the effect of path clutterness and endpoint crowding by parametrically manipulating crowding and clutterness at three different levels: baseline, single level (i.e., one instance of clutter/crowding), and multiple level (i.e., multiple instances of clutter/crowding), while controlling for other factors (Figure 1). We hypothesized that both increased levels of path clutterness and endpoint crowding (multiple level > single level > baseline) in the modified block tapping task will lead to decreased VSWM task performance.

## 2. Methods

This study was pre-registered on OSF (https://osf.io/ypwfx).

### 2.1. Participants

We recruited 71 participants from the Department of Psychology’s research participant pool at Northeastern University’s Boston campus. In accordance with the guidelines of the university’s Institutional Review Board for Human Research, written informed consent was obtained from all participants prior to the study. Participants were remunerated with either a $10 Amazon gift card or course credit as compensation upon completion of the study. The experimental session took around 60 minutes. 11 participants were excluded from the study due to meeting at least one of the following data exclusion criteria:

1. Having vision problems or a physical handicap that prevents one from completing the experimental task;
2. 10 having a response hit rate below 50% under all experimental levels (baseline, single, and multiple) for at least one condition being tested (path clutterness, endpoint crowding).^1^

Thus, our final sample included 60 participants (*Mean*_age_ = 19.88, *SD*_age_ = 1.95, *Range*_age_ = [18, 29]; 32 females, 15 males, 1 other; 12 declined to answer).

### 2.2. Sequential sampling

The final sample size was determined by sequential sampling with a Bayes Factor Stopping Rule (Wagenmakers, Gronau & Vandekerckhove, 2019; Schönbrodt & Wagenmakers, 2018; Rouder, 2014). For every three participants and starting at a sample size of 30, we ran the analyses for our two hypotheses (see *2.5. Analysis Plan* below). We a priori specified that if the relative evidence strength exceeded a threshold of *BF*_10_ = 15 in support of the research hypothesis or *BF*_01_ = 10 in favor of the null for both analyses, then we would stop data collection (noting that the asymmetry in the BF for the stopping rule for the null and alternative hypotheses reflects the known difficulty in gathering evidence for the null (Joessel, Cunningham & Green, 2025)). We also defined a maximal sample size of *N* = 60 where data collection should be stopped regardless of the current evidence strength (since there is no guarantee such a procedure will ever reach the proposed stopping BF values). We ended data collection by hitting the pre-defined maximal sample size. While the Bayes Factor for endpoint crowding exceeded the *BF*_10_ threshold when we initially analyzed the data at *N* = 30, the evidence for the impact of path clutterness remained inconclusive throughout sampling (Figure S2, *Supplementary Materials*).

### 2.3. Materials

The task was programmed in PsychoPy v2025.1.1 (Peirce et al., 2019) and administered through the standalone software installed on a Dell XPS 8950 computer paired with a 24-inch screen (1920 × 1080 resolution and a 60 Hz refresh rate), operating on the Windows 11 system. Circles representing spatial locations in the CBTT had a radius of 35 pixels, presented in white on a grey background. Here we note that circles, rather than squares, as are often employed in CBTTs, allowed for a more precise manipulation and adequate control of the distance between spatial locations. In total, 12 spatial locations were used to provide sufficient flexibility to construct sequences with a moderate difficulty level for young adults (span = 6) while also leaving additional circles available for distractor placements. Participants used the spacebar on a keyboard to progress through task phases and used a mouse to select target items during trials. A chinrest to fix participants’ viewing distance during the experiment was placed 35.6 cm (14 inches) from the computer screen. Therefore, each circle stimulus had a diameter of 3.1° of visual angle.

### 2.4. Design and procedure

Path clutterness was operationalized as defined by Ginsberg, Rinehart & Fielding (2017) as when at least one distractor circle overlapped with the area formed by the most distant lines parallel to the path linking two consecutive target circles in the sequence that still connect these two target circles. A segment could only be cluttered or not cluttered, regardless of the number of elements in the clutter area (Figure 1a). Endpoint crowding was operationalized by placing distractor circles at an edge-to-edge distance closer than 0.85° of visual angle from a target circle (Figure 1b), which is within the expected range to induce visual crowding in the fovea (Lev, Yehezkel & Polat, 2014; Levi & Carney, 2009). For each condition, we designed sequences with three levels (baseline clutterness/crowding, single level clutterness/crowding, multiple level clutternesses/crowdings), varying in the number of paths/endpoints being cluttered/crowded (Figure 1).

All sequences contained six items, typical of the memory span of healthy adults (*M* = 6.2, *SD* = 1.3) found in the traditional CBTT (Kessels et al., 2000). Using target sequences with the same length allowed us to better examine the specific impact of each visual-perceptual condition. To keep trials within each level as uniform as possible, the second-to-last spatial path or the second-to-last circle in the sequence was chosen to be cluttered/crowded in the single-level condition. As such, we preserved the spatial configurations to the greatest extent while achieving a higher level of path clutterness/endpoint crowding. Consequently, the circles serving as distractors could either be part of the target sequence or be non-target items (Figure 1). We created five base sequences (each with a unique circle layout and all three levels of condition) for both the clutterness and crowding. The 10 (two conditions × five layouts) base sequences were then flipped horizontally, vertically, or both horizontally and vertically to generate additional sequence layouts while preserving the key features (see Figure S1, *Supplementary Materials*). This approach resulted in 60 trials (five layouts × three levels × four orientations) to test each condition thoroughly. In total, each participant completed 120 (two conditions × 60 trials) trials of circle sequences presented in a pseudo-random order during the experiment. These 120 trials were divided into 12 blocks, with each containing a balanced number of conditions and levels. Trials manipulating crowding or clutter were interleaved to minimize the timing impact on any experimental condition throughout the session. The order of blocks and trials in each block presented was random for each participant. By the end of the experiment, participants were tested under each level (baseline, single, multiple) 20 times for each of clutterness and crowding. At the beginning of the session, participants also completed five practice trials with feedback to get familiar with the task.

At the beginning of each trial, a fixation cross would appear (1000 ms) at the center of the screen to fix participants’ eye gaze, following was a “watch” text (1000 ms) which prompted them to pay attention to the stimuli that were about to appear: All 12 circular locations were laid out first (1000 ms before highlight), and the target circles were shown sequentially (white circle turning blue), each with a duration of 1000 ms. The inter-stimulus interval was 500 ms (e.g., the current target circle turned back into white for 500 ms before the next target circle was shown in blue). After all six circles in a sequence were shown, a text (“Click and repeat sequence. When done, press SPACE”) would appear at the top of the screen to prompt the participant to start replicating the sequence by clicking on the appropriate circles. As participants responded, the clicked circles turned yellow. Once clicked, participants could not go back and edit their response. The responding phase was not limited in time; once finished, participants pressed the space bar to proceed to the next trial. After completing each block (10 trials), participants were given a 30-second break during which they could move their head away from the chinrest. Upon finishing half of the experiment (six out of 12 blocks), participants were instructed to take a longer break of three minutes outside the experiment room. These scheduled breaks were implemented to help them relax and maintain focus throughout the session. At the end of the experiment, participants were briefly interviewed about their experience and strategy use. They were also asked to complete a demographic survey. Throughout the experiment, the response time of each sequence was measured as the interval between the onset of the response phase and the spacebar press initiating the next trial or the break.

### 2.5. Analysis Plan

A mixed-effects repeated-measures logistic regression was employed for perceptual path clutterness & endpoint crowding independently, with level (baseline, single, multiple) as the fixed effect in the model and a random intercept allowing each subject to have their own baseline performance. Both the frequentist approach and the Bayesian framework were used to interpret the results. Under the Bayesian approach, we compared the evidence in favor of the research hypothesis over the null. We hypothesized that the presence of the perceptual factor (path clutterness/endpoint crowding) would decrease participants’ VSWM performance. Therefore, for the research hypothesis, we modeled performance (log-odds of correctly replicating a sequence) as a linear combination of the log-odds of correct replication under baseline (*β*_0_), change in log-odds compared to baseline with the presence of single clutterness/crowding (*β*_1_ Level*_ij_*_,single_), change in log-odds compared to baseline with the presence of multiple clutterness/crowding (*β*_2_ Level*_ij_*_,multiple_), and a random intercept that captures the individual variation (*u_i_* ~ *N*(0, *σ*^2^_u_)). In the null hypothesis, however, performance is only modeled as a linear combination of the log-odds of correct replication under baseline and the random intercept.

#### Research Hypothesis


1 $$\text{logit}\left[P\left({\text{Y}}_{\text{ij}}=1\right)\right]=\beta_{0}+\beta_{1}\;{\text{Level}}_{\text{ij},\text{single}}+\beta_{2}\;{\text{Level}}_{\text{ij},\text{multiple}}+u_{i}$$

#### Null Hypothesis


2 $$\text{logit}\left[P\left({\text{Y}}_{\text{ij}}=1\right)\right]=\beta_{0}+u_{i}$$

For both perceptual path clutterness & endpoint crowding, a conclusive result with strong evidence would occur if either the Bayes Factor exceeded the predefined threshold in favor of the research hypothesis over the null (*BF*_10_ > 15) or vice versa (*BF*_01_ > 10).

Further analysis of response time (RT) in sequence replication was conducted using a repeated-measures ANOVA after trimming the upper 2% of RTs with each level of clutterness and crowding and applying log-transformation.

## 3. Results

### 3.1. Sequence replication

To examine whether line clutterness and/or endpoint crowding impacted estimates of VSWM, we compared both the hit rate and odds of correct sequence replication of participants’ responses under all three conditions (baseline, single, and multiple) for both path clutterness and endpoint crowding, respectively. Through mixed-effects logistic regression, we examined 1) the coefficient (*β*) of the clutterness or crowding condition (e.g., multiple clutterness) that either increases or decreases the log odds of a correct replication of the sequence given the condition, and 2) the odds ratio (*OR*) of two experimental conditions.

**Figure 2:**
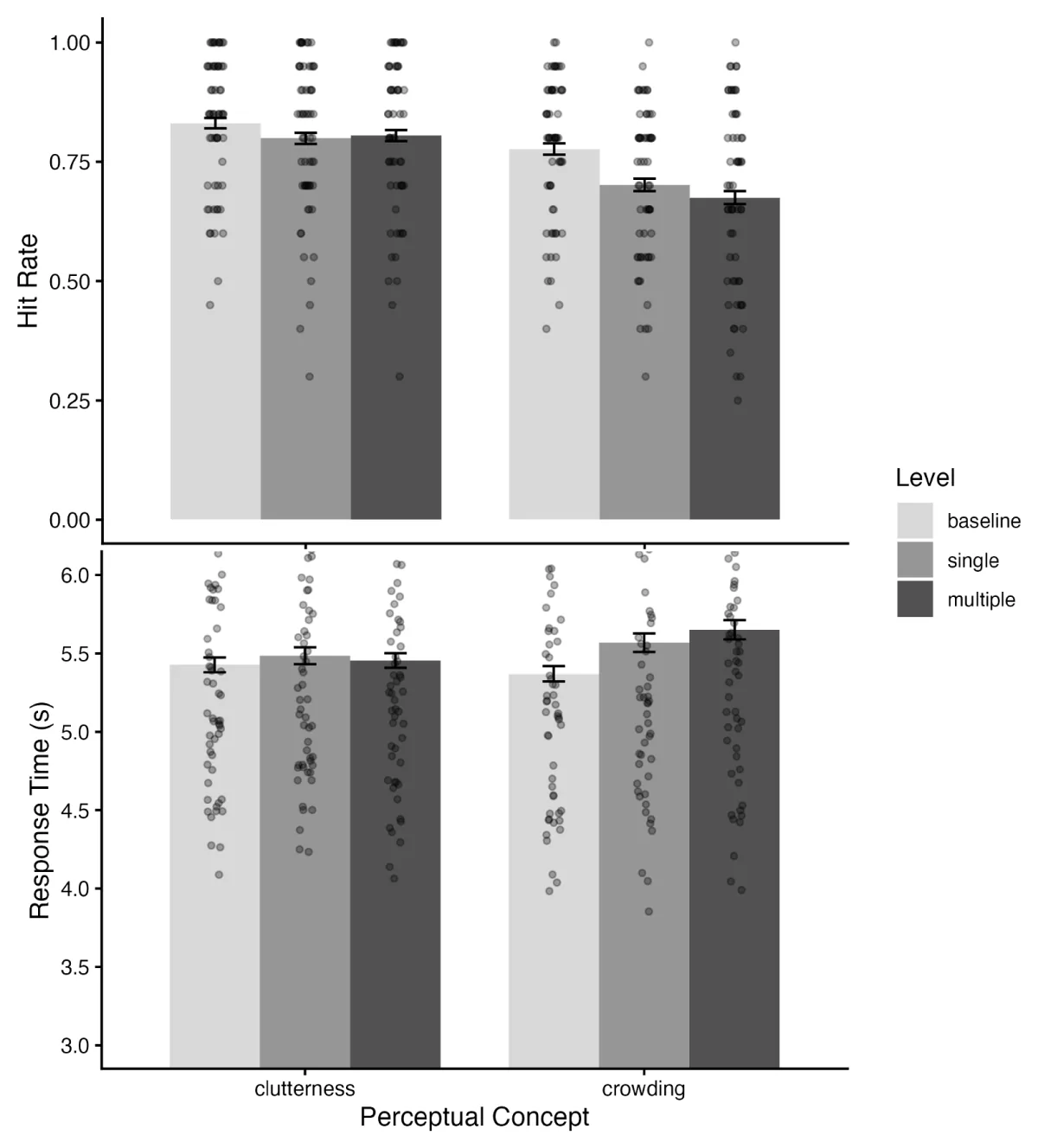
Average hit rate (top) and response time (bottom) by clutterness (left), crowding (right), and by level. ***Note.*** Grey shade represents levels (baseline, single, multiple) for each of clutterness or crowding. Data points represent individual performance data. Error bars show the standard error around the mean.

We expected that VSWM measured by the modified CBTT would vary as a function of path clutterness. However, the results were inconsistent. Under the baseline condition, participants were able to reach a high hit rate (*M* = 0.83, *SE* = 0.01); yet, their hit rate was only slightly lower in single (*M* = 0.80, *SE* = 0.01) and multiple (*M* = 0.81, *SE* = 0.01) clutterness conditions (Figure 2 – top left). Throughout the experiment, baseline demonstrated higher hit rates than the other conditions in the first three blocks, but this pattern diminished in the following blocks (Figure S3, *Supplementary Materials*). Using the frequentist approach, relative to the baseline, presenting sequences in the single clutterness significantly affected participants’ hit rate (log odds of correct sequence replication) in the logistic regression model (*β_1_* = –0.242, *SE* = 0.113, *p* = .031). However, the corresponding post-hoc pairwise comparison between baseline and single conditions was weaker and did not clearly exceed the adjusted significance threshold (*OR*_baseline/single_ = 1.27, *p* = .078). Similarly, compared to the baseline condition, the multiple clutterness condition yielded a marginally significant impact (*β_2_* = –0.200, *SE* = 0.114, *p* = .076) on hit rate in the model-based test but showed no significant difference in the post-hoc test (*OR*_baseline/multiple_ = 1.22, *p* = .178). Post-hoc pairwise comparisons also showed no significant difference between the hit rate under single and multiple clutterness levels (*OR*_multiple/single_ = 1.044, *p* = .920). Rather than indicating statistical inconsistency, the more conservative post-hoc pairwise comparison suggests that the effect of clutterness was modest. Analysis from the Bayesian framework showed similar results (Table S1, *Supplementary Materials*) and indicated anecdotal evidence for the null (*BF*_10_= 0.924, *N* = 60) with omnibus comparison, according to Jeffreys’ Bayes factor cutoff (Jeffreys, 1961). Bayesian directional hypothesis testing with the research hypothesis, on the other hand, provided evidence for a reduction in performance with single-level clutterness (*β_1_* = –0.24, posterior probability = .99, evidence ratio = 71.29) and with multiple-level clutterness (*β_2_* = –0.20, posterior probability = .96, evidence ratio = 23.69). But there was no evidence that multiple clutterness influences performance more than single clutterness (*β* = 0.04, posterior probability = .35, evidence ratio = 0.54). Further, response times (Figure 2- bottom left) showed no significant differences across levels in the path clutterness condition, *F*(1.90, 112.23) = 0.81, *p* = .441, η_p_² = .014. In the single clutterness condition, participants took a longer time identifying the target circle at the end of the cluttered path compared to the other targets (Figure S6, *Supplementary Materials*). In the error analysis, participants did not select more distractors in the clutter zone compared to the other distractors (Figure S8, *Supplementary Materials*). Altogether, this suggests that there is an inconsistent effect of path clutterness on performance. However, further investigation (e.g., with a greater sample size) would be needed to establish that more conclusively.

In the case of endpoint crowding, as hypothesized, participants’ hit rate was highest in the baseline condition (*M* = 0.78, *SE* = 0.01), and was reduced under both single (*M* = 0.70, *SE* = 0.01) and multiple (*M* = 0.68, *SE* = 0.01) crowding conditions (Figure 2 – top right). The baseline advantage persisted throughout the experiment (Figure S4, *Supplementary Materials*). Consistent with this pattern, logistic regression revealed significantly lower log odds of correct sequence replication relative to baseline for both the single crowding condition (*β_1_* = –0.432, *SE* = 0.098, *p* < .0001) and the multiple crowding condition (*β_2_* = –0.570, *SE* = 0.097, *p* < .0001). Post-hoc pairwise comparisons confirmed these effects, with both crowding conditions yielding significantly lower odds of correct replication than baseline after contrast-based adjustment (*OR*_baseline/single_ = 1.540, *p* < .0001; *OR*_baseline/multiple_ = 1.769, *p* < .0001). Although the direct contrast between the single and multiple crowding conditions did not reach significance in post-hoc testing (*OR*_multiple/single_ = 0.870, *p* = .293), the regression coefficients showed a clear monotonic pattern, with a more negative effect for multiple than for single crowding. Bayesian analyses for omnibus comparison converged with these findings, indicating substantially lower log odds of correct sequence replication under crowding conditions relative to baseline and providing extreme evidence for the alternative hypothesis (*BF*_10_ > 100; Table S1, *Supplementary Materials*; Jeffreys, 1961). In Bayesian directional hypothesis testing with the research hypothesis, the single-level crowding (*β_1_* = –0.43, posterior probability ≈ 1, evidence ratio → ∞) and the multiple-level crowding (*β_2_* = –0.57, posterior probability ≈ 1, evidence ratio → ∞) both strongly and negatively impacted performance. Multiple-level crowding also produced a further reduction in performance relative to the single-level condition (*β* = –0.14, posterior probability = .93, evidence ratio = 13.41), although the evidence was not as strong. Consistent with these effects, response times differed significantly across levels, *F*(1.85, 109.35) = 9.82, *p* < .001, η_p_² = .143, with slower responses in both the single level, *t*(59) = 2.865, *p* = .016, and the multiple level, *t*(59) = 4.815, *p* < .0001, relative to the baseline (Figure 2- bottom right). In the single crowding condition, participants took a longer time identifying the circle preceding the crowded target, but did not take a longer time identifying the target being crowded (Figure S7, *Supplementary Materials*). Importantly, in the error analysis, at the crowded circle, participants selected more flankers (distractors that formed crowding) compared to other distractors (Figure S8, *Supplementary Materials*). Together, these demonstrate that the impact of endpoint crowding on task performance is not only consistent but also directionally ordered.

### 3.2. Learning Effect

Because recent work has demonstrated that learning can play a major role in performance on even reasonably short cognitive assessment measures (Cochrane & Green, 2021), we next performed a series of exploratory analyses to further assess the impact of crowding and clutterness and the extent to which they might be affected by learning over the course of the experiment. If certain conditions of visuospatial layout were more readily detectable and learned, then participants might be able to inhibit the influence of those distractors, and the extent to which performance reflects perceptual contamination would be reduced. Here we collapsed the data into four experimental time points (T1, T2, T3, T4) and conducted repeated-measures ANOVAs with follow-up pairwise comparisons.

**Figure 3:**
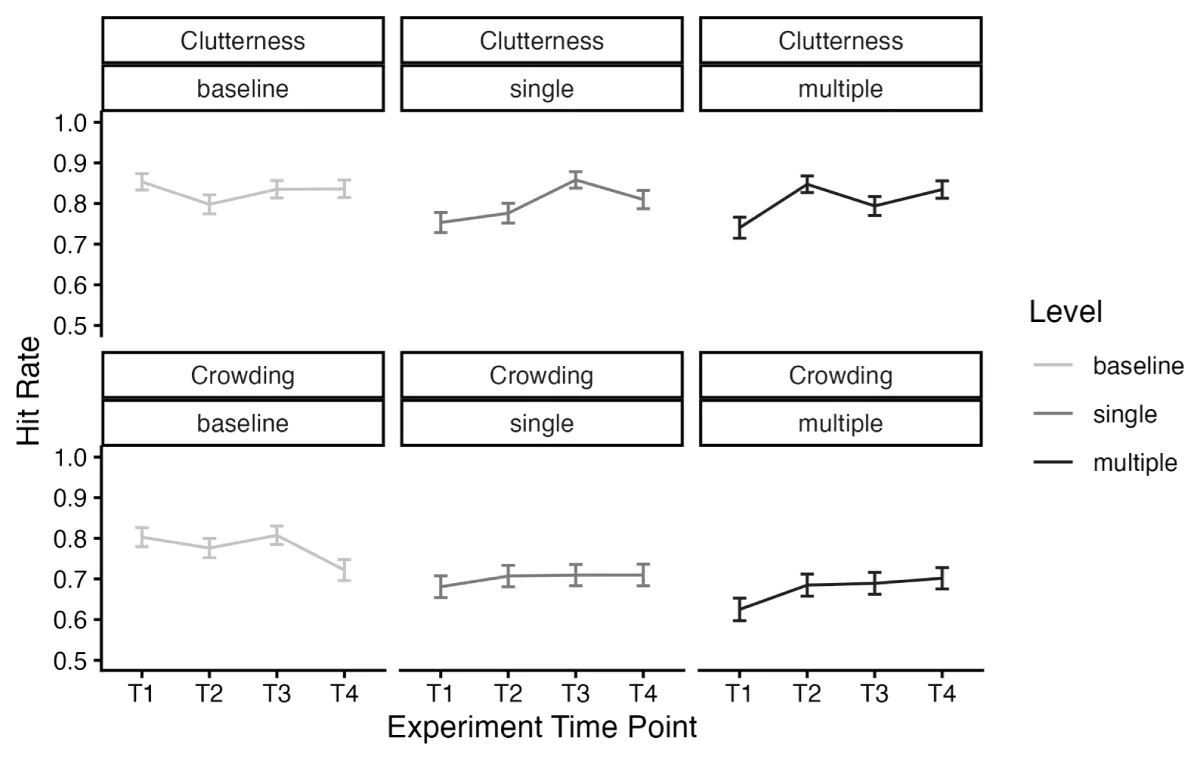
Average hit rate with standard error across experiment time points. ***Note.*** The experiment session consists of 12 blocks with 10 trials each. Three blocks are collapsed into one time point for illustration purposes. Grey shade represents the level of clutterness or crowding.

Under path clutterness, hit rate fluctuated across time points in some conditions (Figure 3, top). Specifically, under single level, performance differed between T1 and T3 (*t*(59) = –3.03, *p* = .021). Under multiple level, differences were observed between T1 and T2 (*t*(59) = –3.17, *p* = .015) and between T1 and T4 (*t*(59) = –3.04, *p* = .021). In all, performance did not improve consistently across time, but there did appear to be some learning gains that allowed multiple clutterness to eventually reach a performance level comparable to that of the baseline.

In contrast, we did not observe any timepoint-related hit rate difference under single (*F*(3, 118) = 0.21, *p* = .892) and multiple (*F*(3, 118) = 1.16, *p* = .327) levels of endpoint crowding. Hit rates consistently remained below baseline (Figure 3, bottom), suggesting a robust and persistent contamination effect that was largely resistant to learning. Although a modest effect of time was observed under baseline crowding (*F*(3, 188) = 2.75, *p* = .044), adjusted post-hoc comparisons did not reveal a significant difference between any pairs of time points. This pattern suggests there was some fatigue throughout the experiment, but not to an extent that significantly affected performance.

## 4. Discussion

The primary goal of this study was to test the hypothesis that visual contaminants would impact estimates of Visual Spatial Working Memory performance in the context of a modified CBTT. To accomplish this, we placed distractors on the virtual path of temporally adjacent spatial targets to induce path clutterness and placed distractors in proximity to the spatial targets themselves to induce endpoint crowding. As hypothesized, we found that VSWM performance was impacted by endpoint crowding; however, we found a limited and inconsistent impact of line clutterness.

With endpoint crowding, the task hit rate was reduced by 10–13%, which was supported by strong evidence under the Bayesian framework (Table S2, *Supplementary Materials*). These results are consistent with data from other visual-spatial tasks. For example, in visual search, crowding, operationalized as item clusters, also resulted in a lower rate of target hits than in the uncrowded condition (Wolfe et al., 2024). In Wolfe and colleagues’ experiment design (2024), the target was only hidden in the clusters 50% of the time, so that participants could not rely on the cluster feature during search. In our block tapping task, even though crowding spots always contained a target, we did not observe any significant learning effect in this condition, so this did not help participants identify the targets. In both experiment contexts, targets embedded in clusters/crowding spots had a higher chance of being missed. Whether the influence of the crowding feature on the visual search task and the visuospatial working memory task was a result of incomplete processing in the useful field of view (Wolfe et al., 2024), lowered visuospatial attention (Kewan-Khalayly & Yashar, 2022), variable-precision dynamics in working memory (Ma, Husain & Bays, 2014), or other mechanisms, is worth future investigation.

On the other hand, evidence of any impact of path clutterness on the difficulty of the VSWM task was more equivocal and did not firmly support the hypothesis put forward by Ginsberg, Rinehart & Fielding (2017). In contrast to our study, in Ginsberg and colleagues’ study, clutterness was intermixed with other visuospatial features (e.g., path crossings). Importantly, directional hypothesis testing under both frequentist and Bayesian frameworks provided evidence for an effect of clutterness, although the magnitude of this effect was weaker than that observed for crowding. In contrast to the significant slowing in sequence replication response times associated with crowding, response times remained comparable across levels of clutterness. Further, we did observe a larger path clutterness effect at the early stage of the session that diminished across the session (Figure S3, *Supplementary Materials*). Thus it may be the case that there are initial impacts of path clutterness that participants are able to adapt to over time. Together, these findings suggest that while clutterness may influence VSWM performance, at least under the conditions employed in our study, its effects are not as strong as those of crowding. That said, other operationalizations of clutter may have stronger effects, and it is plausible that stronger line-clutterness effects may occur when blocks actually interrupt the line paths rather than just abutting them. Further research will be required to address the extent to which greater disruptions of line-path may provide more perceptual contamination and to clarify the conditions in which people may adapt to clutterness effects on estimates of people’s visuospatial working memory.

We note that our study also has limitations. For example, we only used sequences with six circles. The influence of line clutterness and endpoint crowding might be different when people are asked to complete longer sequences that more completely challenge their VSWM limit. At higher cognitive loads, the working memory system is more likely to reveal its limitations, such that the precision of item features (e.g., location in CBTT) will trade off with the span size (Ma, Husain & Bays, 2014). Second, our experiment samples were limited to college students, with a narrow age range. Third, current experiments underscore variations in VSWM task design, but the dynamic internal experiences of individuals also interact with the measurement of VSWM. For example, Gonthier (2021) investigated people’s strategic processes in visuospatial short-term memory (VSSTM) and suggested that researchers either systematically consider their strategic processes during VSSTM measurement or limit/remove the use of strategies and pinpoint the target process. In fact, an earlier study on CBTT found that participants recruited different strategies, including “linking targets with imagery lines”, “verbally recoding the spatial locations”, and “finger-tracing the sequential locations” for this same task (Patt et al., 2014). These strategies lead to differences in the characteristics of the task engagement between participants and raise questions such as: Do people use imagery lines more subject to the influence of path clutterness? Does verbal recoding turn the CBTT into a complex working memory task (dual task requirement)? How visuospatial designs trigger and interact with strategy use is worth further investigation. Whether a pure measurement of VSWM exists remains an open question. By understanding the impacts of these perceptual contaminants, we can control them in experimental settings, assign weights to trials with varying loads (Conway et al. 2005), and more fairly compare across study results. Improved measurement requires recognition of the multidimensional nature of VSWM.

Overall, our study contributes to the understanding of the impacts of task configurations on VSWM assessment, especially in a computerized task environment. More broadly, our findings support that the lower-level visual component plays an important role in the VSWM assessment tasks (Adam, Rademaker & Serences, 2022; Serences, 2016). This aligns with the view that working memory is a distributed system, from the perceptual details originating in the sensory cortex for early maintenance to information abstraction with the prefrontal areas for task-relevant actions (Christophel et al., 2017). Accordingly, when designing and interpreting assessment tools such as the Corsi Block Tapping Task, it is essential to consider the underlying cognitive processes. Differences in task procedures and configurations may significantly impact outcomes and limit comparability across studies. Although such perceptual influences do not invalidate established visuospatial working memory measures, they may nonetheless contribute systematic variability to performance, particularly in modified or experimentally customized task designs. The contribution of perceptual factors may be especially important when assessments rely on a relatively small number of trials or when performance is compared across groups, as unmodeled perceptual characteristics could differentially influence task difficulty and potentially affect the interpretation of group differences. Therefore, explicit consideration of how perceptual factors influence estimates of visuospatial working memory can improve experimental control, measurement precision, and cross-study comparability.

## Additional File

The additional file for this article can be found as follows:

10.5334/joc.517.s1 Supplementary Materials. Figures S1 to S8 and Tables S1 to S2.

## Data Availability

Data and scripts are available on OSF: https://osf.io/ypwfx.

## Note

College students unable to perform at a 50% hit rate for any level of the task are likely not complying with task instructions or appropriately engaging with the task.
