# Supplementary material for: Perceptual Contaminants to Assessments of Visuospatial Working Memory: The Influences of Path Clutterness and Endpoint Crowding
Source: J Cogn. 2026 Aug 25;9(1):44. doi: 10.5334/joc.517 (PMC13523809; doi:10.5334/joc.517)
Supplement: Supplementary file 1 [file joc-9-1-517-s1.pdf]

Supplementary Materials

for

Perceptual Contaminants to Assessments of Visuospatial Working Memory: The Influences of Path Clutterness and Endpoint Crowding

Zechun Zhao[a], Freya Joessel[b], C. Shawn Green[b], Susanne M. Jaeggi[a], Aaron R. Seitz[a]

[a] *Department of Psychology, Northeastern University, Boston, USA*

[b] *Department of Psychology, University of Wisconsin-Madison, Madison, USA*

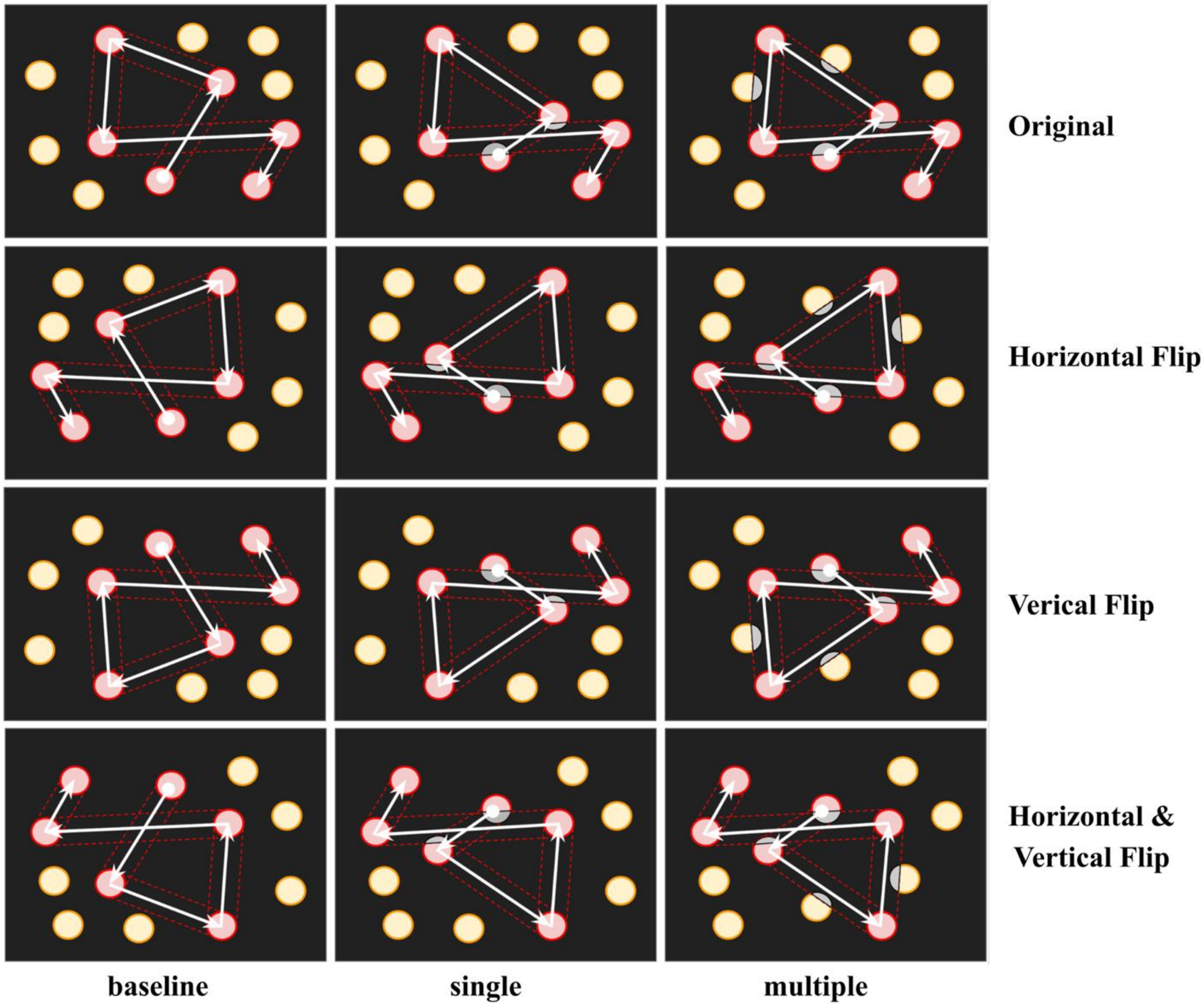

Figure S1-A. Example of Path Clutterness with three levels and four orientations. Sets of sequence examples with four different layouts generated by flipping the entire task field. Pink circles are targets in the sequence; Yellow circles are distractors; Grey area shows overlap with the clutter zone. Solid white line with arrow shows direction of the sequence; Red dashed lines form the clutter zone of each sequence path; All lines are invisible, and there is no color differentiation between circles during the experiment.

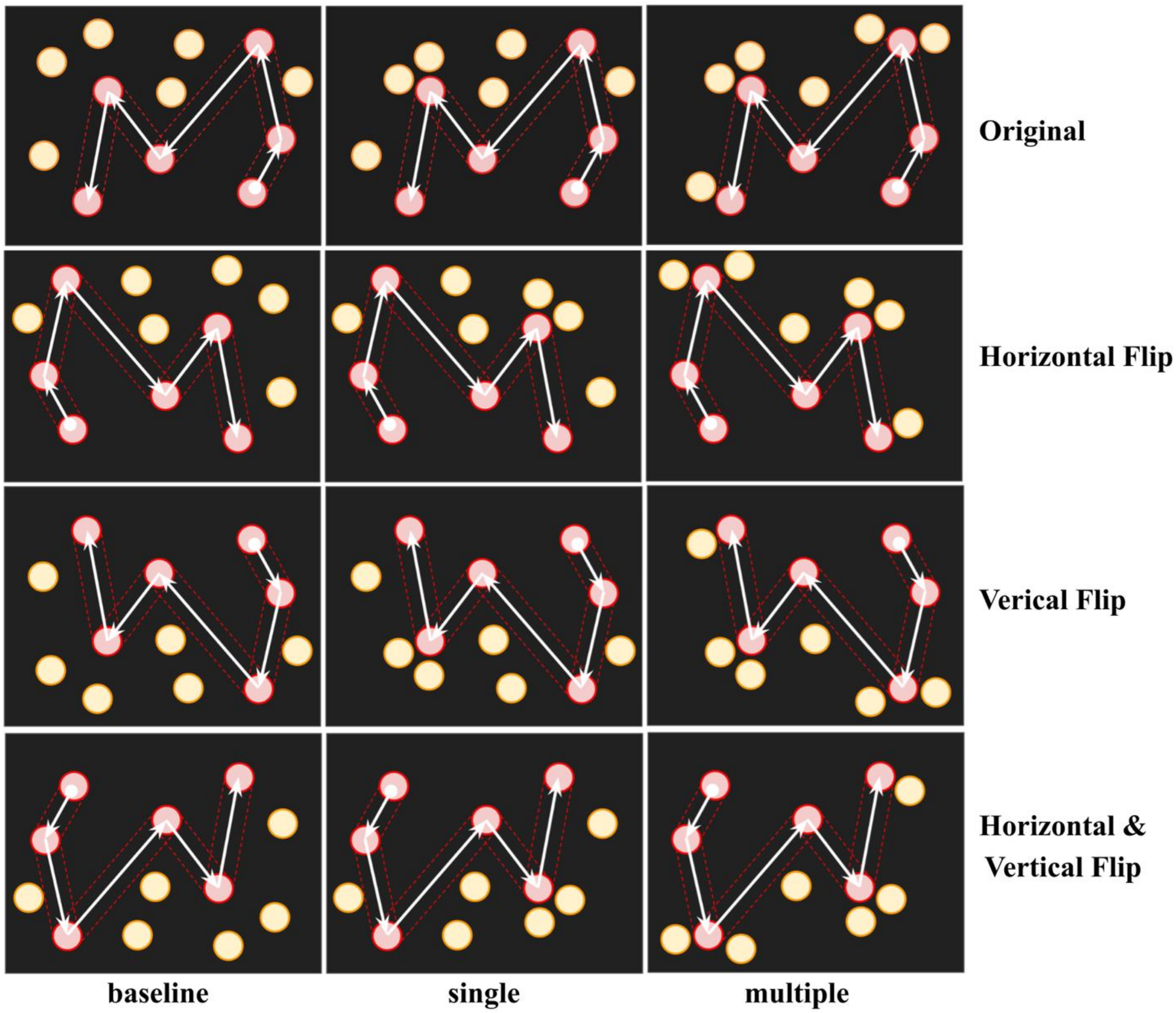

Figure S1-B. Example of Endpoint Crowding with three levels and four orientations. Sets of sequence examples with four different layouts generated by flipping the entire task field. Pink circles are targets in the sequence; Yellow circles are distractors. Solid white line with arrow shows direction of the sequence; Red dashed lines form the clutter zone of each sequence path; All lines are invisible, and there is no color differentiation between circles during the experiment.

Table S1

Table S1. Bayesian logistic regression posterior parameter estimation

Condition	Level	Estimate	Est.Error	Q2.5	Q97.5
**Path Clutterness**	Intercept	1.97	0.18	1.63	2.33
	Single	-0.24	0.11	-0.47	-0.03
	Multiple	-0.20	0.11	-0.43	0.02
**Endpoint Crowding**	Intercept	1.40	0.13	1.16	1.66
	Single	-0.43	0.10	-0.62	-0.24
	Multiple	-0.57	0.10	-0.76	-0.38

Note. Estimation of single and multiple level beta relative to the baseline (intercept).

Table S2

Bayes Factor along sequential sampling checkpoints for both Path Clutterness and Endpoint Crowding

**Condition**	BF_{10}										
	N = 30	N = 33	N = 36	N = 39	N = 42	N= 45	N = 48	N = 51	N = 54	N = 57	N = 60
**Path Clutterness**	0.23	0.20	0.19	0.26	0.38	0.33	0.37	0.43	0.70	0.63	0.92
**Endpoint Crowding**	163.10	180.43	1403.10	3295.79	6235.67	4617.11	5892.76	9871.64	1837697.31	8643229.77	5878612.86

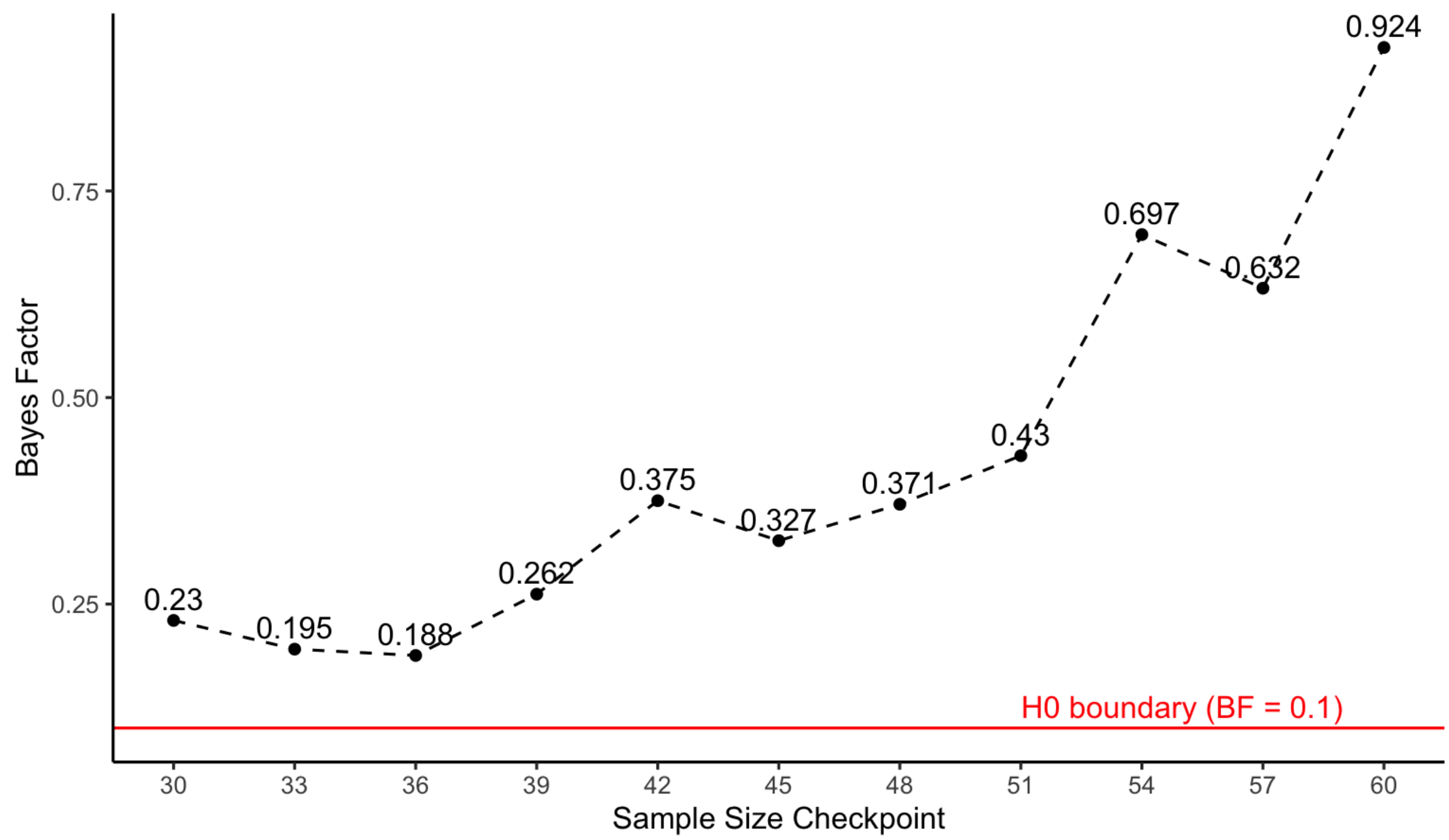

Figure S2. Path Clutterness: BF_{10} along the sequential sampling checkpoints. The Bayes factor represents the relative strength of the alternative versus the null hypothesis. Red annotation shows the boundary in favor of the null hypothesis to stop data collection at. The boundary in favor of the alternative hypothesis (*BF* = 15) to stop data collection is not shown in the figure.

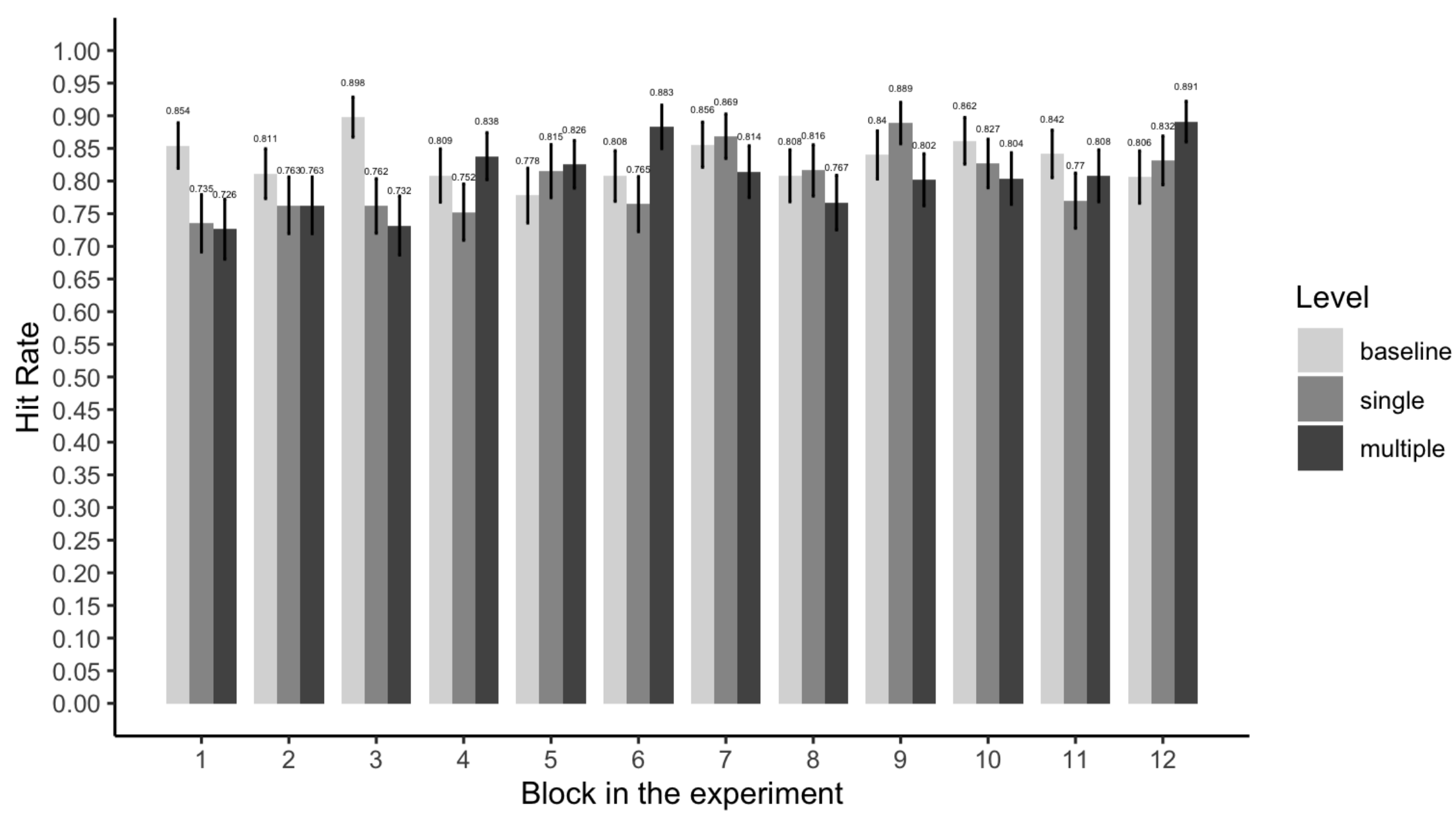

Figure S3. Path Clutterness: Hit Rate at each level across blocks in the experiment. Color shades represent sequence level.

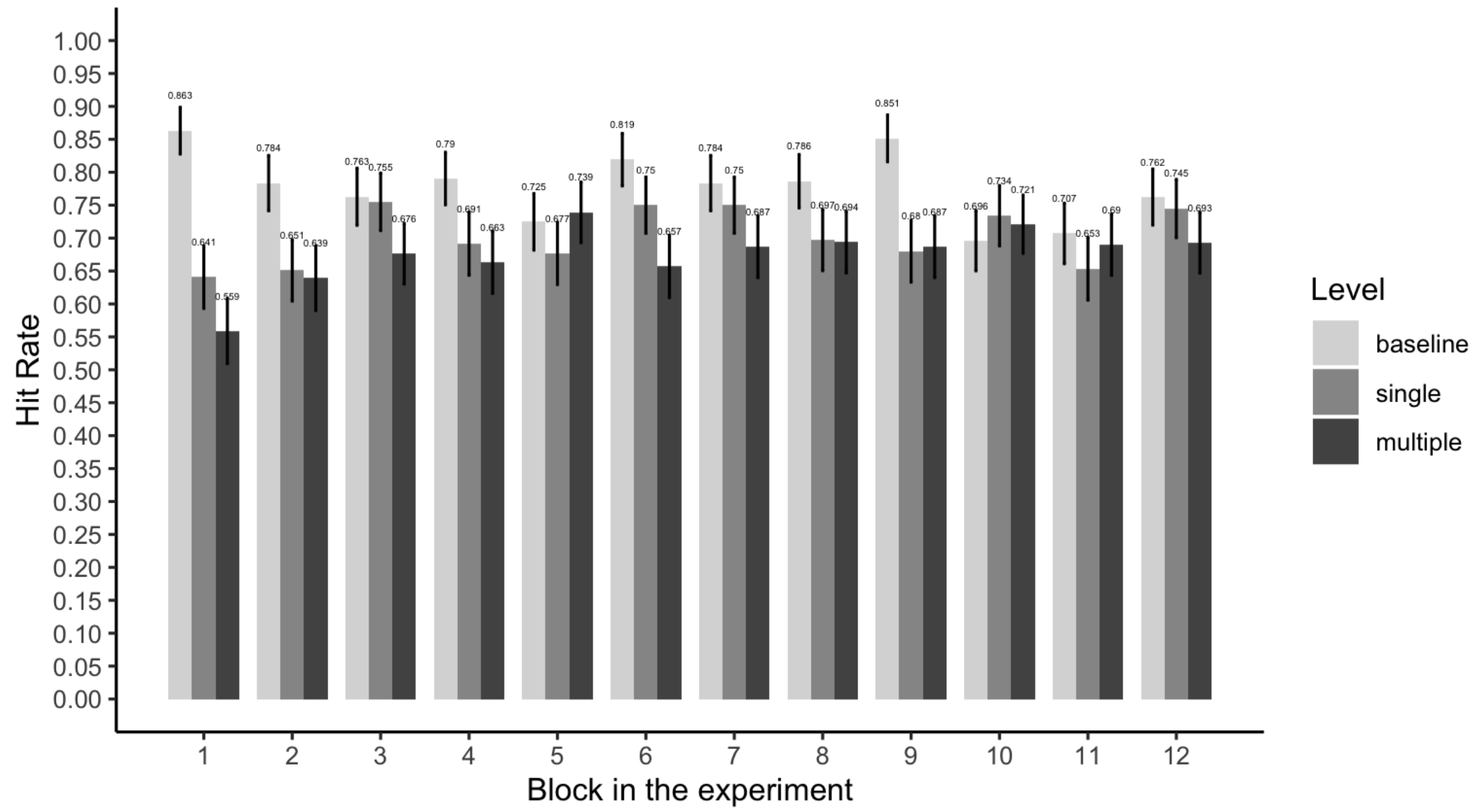

Figure S4. Endpoint Crowding: Hit Rate at each level across blocks in the experiment. Color shades represent sequence level.

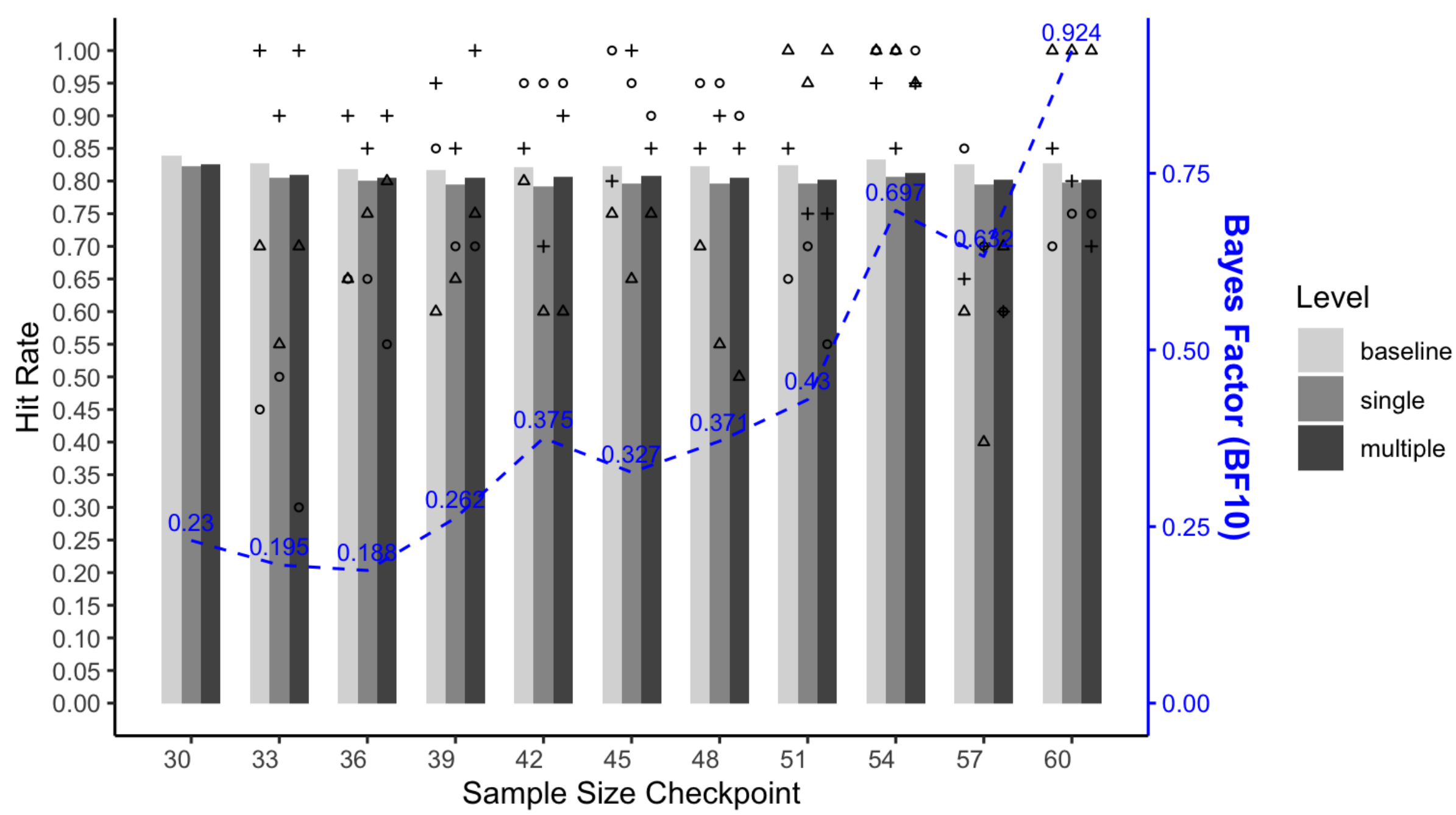

Figure S5. Path Clutterness: Link Bayes Factor change with new subjects' data at each checkpoint. Color shades represent sequence level; Bars represent existing subjects; Points represent new subjects at each checkpoint (individuals identified by shape); Blue annotations map the evolution of the Bayes Factor with hit rate data.

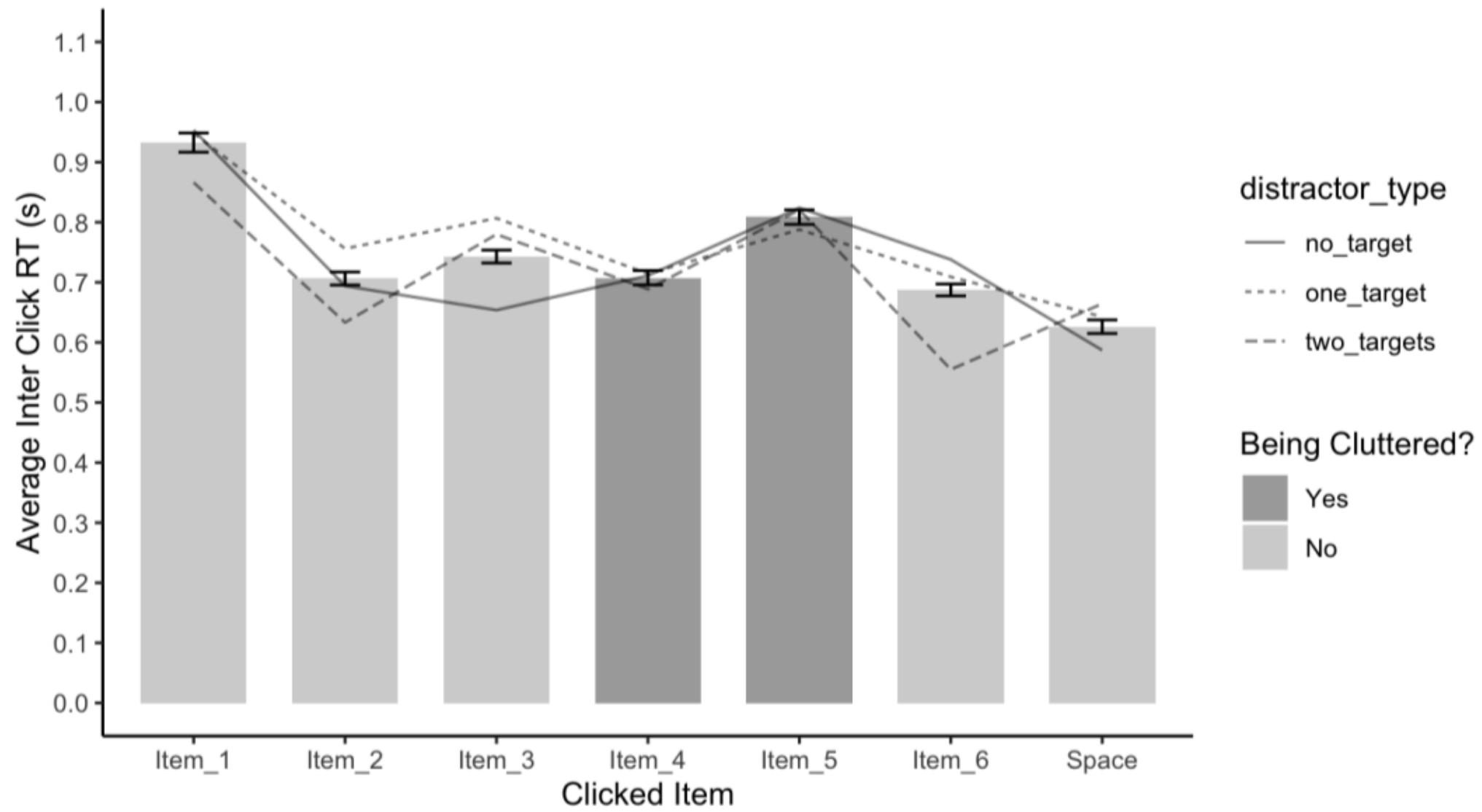

Figure S6. Path Clutterness (single level): Inter-Click Response Time. Time spent between item actions in seconds, averaged across participants and trials. Response time for item 1 is the interval between when participants enter the response phase and when they click the first item. Response time for the space bar is the interval between the participants' last click and their action of pressing the space bar to proceed. Grey shade indicates whether the item is being cluttered. Line type shows the number of distractor circles that were targets in the sequence.)

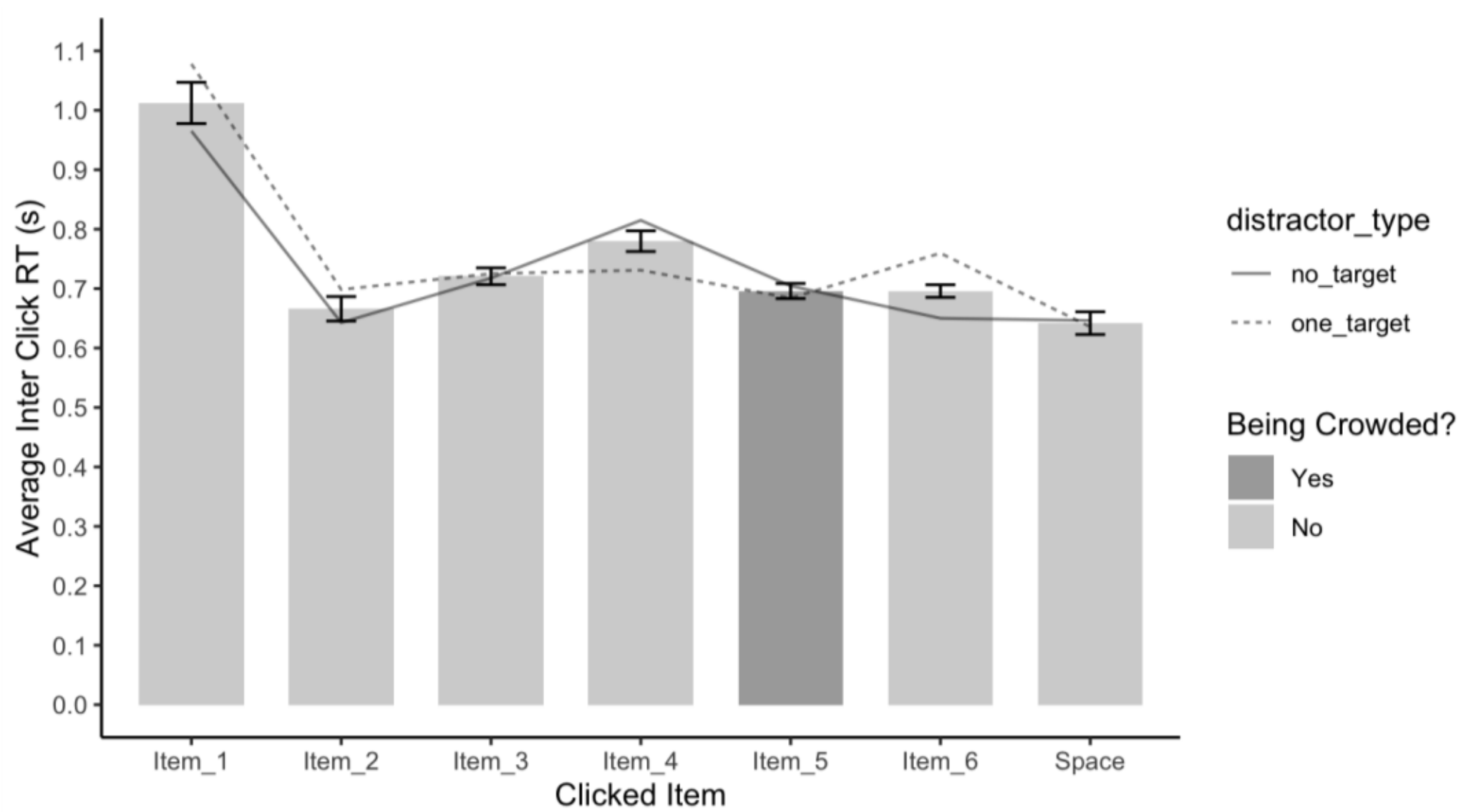

Figure S7. Endpoint Crowding (single level): Inter-Click Response Time. Time spent between item actions in seconds, averaged across participants and trials. Response time for item 1 is the interval between when participants enter the response phase and when they click the first item. Response time for the space bar is the interval between the participants' last click and their action of pressing the space bar to proceed. Grey shade indicates whether the item is being crowded. Line type shows the number of distractor circles that were targets in the sequence.)

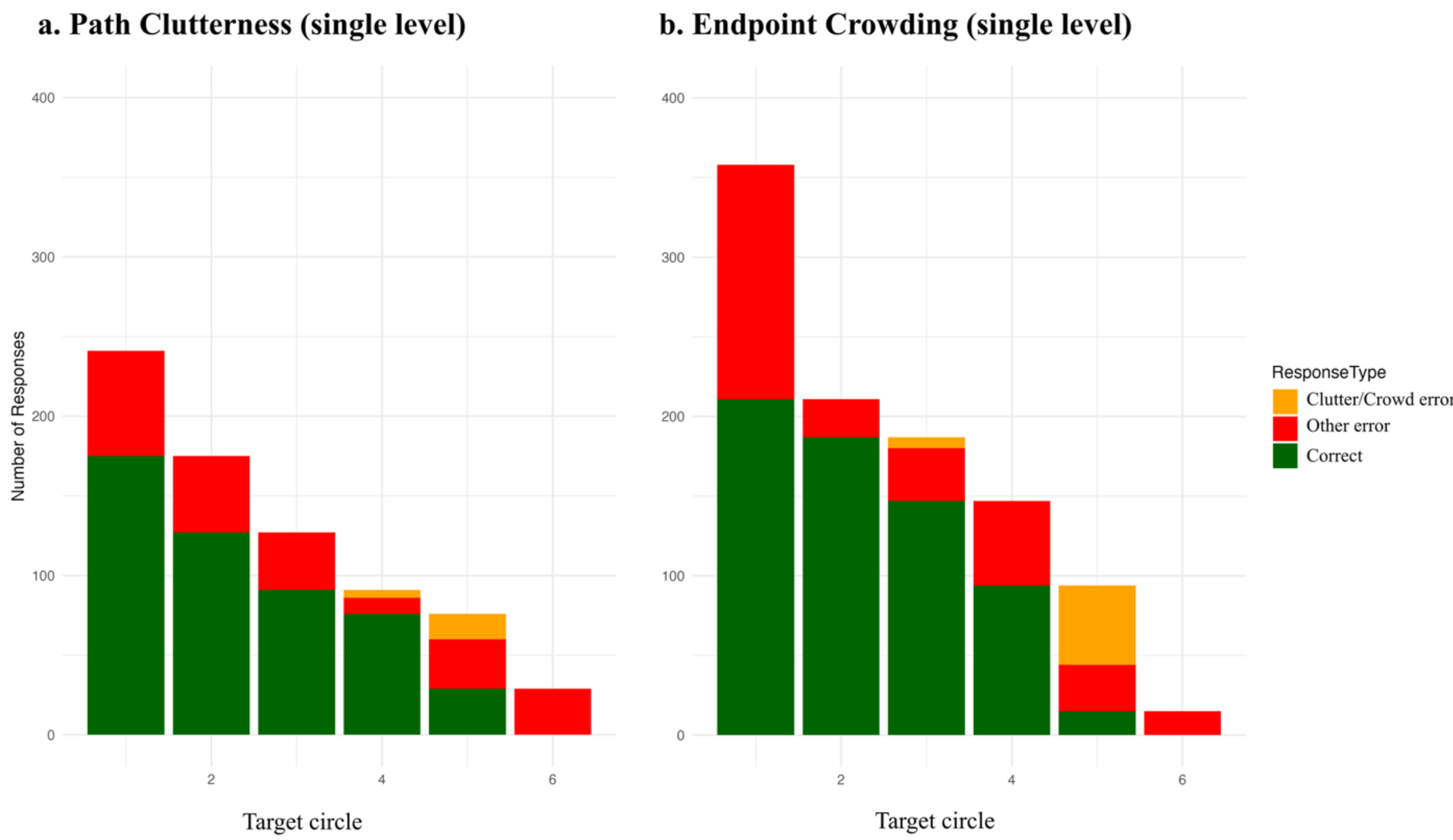

Figure S8. Error Analysis (single level): Stacked Bar Plot of Response Type. For each target circle in the sequence, the stacked bar shows the breakdown of response types. Orange color indicates an error made by selecting the circles in the cluttered zone/formed crowding; red color indicates an error made by selecting any other non-target circles; green color indicates a correct selection. Responses are summarized cumulatively (e.g., if a response is incorrect at the first target circle, then it is excluded from the rest of the target circle analysis). Responses in the plot are all error responses.
